# Treating Penile Cancer in the Immunotherapy and Targeted Therapy Era

**DOI:** 10.1155/2019/8349793

**Published:** 2019-03-25

**Authors:** Gavin Hui, Sanaz N. Ghafouri, John Shen, Sandy Liu, Alexandra Drakaki

**Affiliations:** UCLA Medicine Education Office, RRUCLA Medical Center, 757 Westwood Plaza, Suite 7501, Los Angeles, CA 90095-7417, USA

## Abstract

While localized penile cancers are typically treated surgically and metastatic penile cancers benefit from standard chemotherapy, there have been studies on the horizon demonstrating immunotherapy as a novel approach to metastatic penile cancers that have failed standard chemotherapy. We report a case series of two patients who improved on immunotherapy after progressing with standard chemotherapy regimens. The first case describes a 64-year-old male with a penile mass and significant lymphadenopathy who had surgical resection and adjuvant chemotherapy prior to continued disease progression. He was started on anti-EGFR treatment and improved initially, but he eventually had progression of disease. The second case describes a 79-year-old male with a penile mass who was treated with surgical resection and started on adjuvant chemoradiation, but he developed recurrence and nodal involvement. Therefore, second-line therapy of the PD-L1 inhibitor was started in this patient. There were no available clinical trials for penile cancer patients who progressed beyond the standard surgical therapy and chemotherapy.

## 1. Introduction

In developed countries, penile cancer is considered to be a rare neoplasm that typically affects elderly males. In the U.S., there are about 2300 new cases and 400 deaths from penile cancer annually [1]. Risk factors include phimosis, HPV, HIV, smoking exposure, and lack of circumcision that leads to poor body hygiene. More than 95% of penile cancers are classified histologically as squamous cell carcinomas, while the remaining include adenocarcinoma, melanoma, sarcoma, basal cell carcinoma and lymphoma [2]. The treatment and prognosis depend on the stage of the disease and in particular, the presence and extent of lymph node involvement.

While primary penile tumors can be treated with surgical excision, laser ablation, local radiation, and partial or total penectomy, more advanced tumors, such as those with recurrence or metastasis, benefit from chemotherapy [2]. Penile cancer can spread through the lymphatic system to the inguinal, pelvic, and retroperitoneal lymph nodes [3]. According to National Comprehensive Cancer Network (NCCN) guidelines, penile cancers with unfavorable features such as bulky, fixed lymph nodes, greater than 4 cm in size, portend poorer prognosis and oftentimes warrant neoadjuvant chemotherapy. Evaluation includes fine needle aspiration (FNA) followed by excisional biopsy if FNA results as negative. If either of these biopsies are positive, neoadjuvant chemotherapy is recommended with subsequent inguinal and pelvic lymph node dissection.

The standard neoadjuvant regimen is TIP, which consists of four cycles of paclitaxel, ifosfamide, and cisplatin [4]. According to the phase II study by Pagliaro et al., patients with metastatic penile cancer showed meaningful clinical responses when given neoadjuvant TIP. Of the thirty men who received TIP chemotherapy, 15 (50%) of them had objective response. Nine (30%) of them were alive and free of disease recurrence at median follow-up of 34 months [5]. An alternative regimen is TPF, and consists of docetaxel, cisplatin and fluorouracil. Following neoadjuvant therapy, patients with responses are recommended to undergo surgical resection. However, there are very few standardized treatments for those with continued tumor progression or metastatic disease beyond the TIP or TPF regimen.

Tumor progression after chemotherapy indicates very poor prognosis, with median overall survival (OS) less than 6 months [6]. Therefore, basket clinical trials with targeted therapies for molecular markers such as epidermal growth receptor factor (EGFR), programmed death-1 (PD-1), and programmed death-1 ligand (PD-L1) are imperative in these circumstances. EGFR is a transmembrane tyrosine kinase receptor involved in many cell functions, and it is found to be commonly expressed in penile carcinomas [7]. This suggests the EGFR pathway plays a significant role in penile carcinogenesis, and the literature has shown response in penile cancer patients treated with anti-EGFR monoclonal antibodies [7, 8]. There are currently several studies and clinical trials on epidermal growth factor receptor (EGFR) targets, such as panitumumab and cetuximab.

Immunotherapies targeting the PD-1 and PD-L1 pathways, important for escaping the immune response by tumors, are another promising immunotherapy targets in penile cancer. Udager et al. reported frequent PD-L1 expression (62%, 23/37) in penile cancer [9]. PD-L1 is a ligand that binds to PD-1, an inhibitory T cell surface receptor, to promote self-tolerance and suppress T cell activation. PD-1 inhibitor drugs act by preventing tumor-expressed PD-L1 from suppressing immune response. Two PD-1 inhibitor drugs are nivolumab and pembrolizumab, and they are both IgG4 subclass antibodies that block interaction of PD-1 with PD-L1 [10]. IgG1 antibodies that bind to PD-L1 to inhibit PD-1 contact include atezolizumab, avelumab, and durvalumab. There are currently no large studies or data regarding PD-L1 immunotherapy. However, clinical trials are ongoing.

In this case series, we present two patients with penile cancer metastatic to regional lymph nodes. In both cases, the patients ultimately progressed on chemotherapy, and they received compassionate use of an EGFR inhibitor and a PD-1 inhibitor.

## 2. Case One

A 64-year-old male presented with a two month history of difficulty urinating and was found to have a fungating penile mass involving 50% of his penis. The mass was hard and fixed and extended from the glans proximally up the shaft. He also had bilateral palpable inguinal lymphadenopathy. There were no associated constitutional symptoms. Given there was a high suspicion for malignancy, the patient underwent partial penectomy within a month of presentation. Biopsy results confirmed a pT2 tumor with invasive keratinizing squamous cell carcinoma, poorly differentiated, and tumor size of 5 × 4 × 2.5 cm, with corpus spongiosum and lymphovascular involvements.

Following the procedure, the patient had PET-CT for staging, and imaging revealed enlarged hypermetabolic bilateral axillary lymph nodes concerning for metastatic disease. In addition, there was a large centrally necrotic lymph node conglomerate in his left groin that had increased FDG avidity. The patient had left inguinal and bilateral pelvic lymph node dissections revealing metastatic squamous cell carcinoma in multiple lymph nodes. The left inguinal mass was also found to be metastatic well-differentiated SCC. His diagnosis was staged at T2N3M0.

After his surgical procedures, patient was started on adjuvant chemotherapy. He began first line chemotherapy with paclitaxel, ifosfamide, and cisplatin (TIP). He underwent 4 cycles of TIP but eventually developed disease progression on repeat imaging. At this point, the patient was started on cetuximab given EGFR amplification on tumor analysis with the FoundationOne testing platform. However, the patient had an allergic reaction to cetuximab, so his treatment was changed to panitumumab. The patient had stable disease and a progression-free survival of 6 months with anti-EGFR treatment, which is clinically significant given that this treatment was given in the second-line setting for an aggressive tumor type that other than chemotherapy there is no other approved drug to date.

The patient was ultimately started on the PD-1 inhibitor nivolumab. He had initial response to immunotherapy followed by stable disease, so he had a disease control rate of an additional 6 months with this investigational agent at that time. Ultimately, he was placed on hospice and passed away two years from the the time of diagnosis.

## 3. Case Two

A 79-year-old male with longstanding history of advanced prostate cancer on androgen deprivation therapy presented to his urologist after noticing a mass on the tip of his urethral meatus. A subsequent biopsy of the mass was positive for SCC, and the patient underwent partial penectomy and lymph node dissection that revealed positive right inguinal lymph nodes (three out of seven) revealing pathologic T2N2M0 disease. He received adjuvant chemotherapy by extrapolating data of its benefit when given in the neoadjuvant setting. The standard TIP regimen was not pursued given patient's concern for side effects. The patient proceeded with alternative plan of chemoradiation with 5 weeks of weekly low dose carboplatin and paclitaxel. In addition, he received radiation with a total dose of 5000 cGy over 25 fractions to the right inguinal region. However, the patient developed disease recurrence with nodal involvement nine months later. On restaging CT imaging, the patient was found to have new involvement of the left pelvis. A nodal conglomerate measuring 31×58 mm with central necrotic change was identified in the left inguinal region.

Given the patient's age, performance status, and local recurrence of disease, he was started on therapy with chemoradiation with curative intent one month later. Treatment with an additional round of chemoradiation with low dose carboplatin and paclitaxel was given for 5 weeks. He had radiation with a total dose of 5000 cGy over 25 fractions to the left pelvic region. He had stable disease with chemoradiation, but he eventually developed disease progression within a year from the end of chemotherapy. At that point, he was considered for second-line therapy with the PD-L1 inhibitor atezolizumab. After being on atezolizumab for approximately 2 years, he developed biopsy-proven bullous pemphigoid, an immune-mediated toxicity of the skin that has been described with those agents. A restaging scan at approximately 2 years showed near complete response, so patient has been placed on treatment holiday at the time of this report. He was started on prednisone 1 mg/kg per immune-mediated management guidelines and had quick resolution of his blistering symptoms [11].

## 4. Discussion

The standard neoadjuvant regimen of TIP, consisting of paclitaxel, ifosfamide, and cisplatin, has been found to be one of the most efficacious regimens for patients with penile cancer. In a study of 61 patients, 54 (90%) of them received chemotherapy with TIP. 39 (65%) of these patients had either partial or complete response to the treatment. This study showed that about 50% of patients with response to treatment who also have consolidative lymphadenectomy remained alive at 5 years [4]. However, there are very few standardized treatments for patients with continued disease progression after the standard neoadjuvant treatment. Therefore, there is an unmet need to identify other therapeutic options that could include either targeted therapies or immune checkpoint inhibitors like those that were offered to our patients.

The overexpression of epidermal growth factor receptor (EGFR) is frequently observed in epithelial cancers, including penile SCC [12]. A study of penile SCC case series showed EGFR amplification in 4 (20%) of 20 patients. Furthermore, heterogeneous EGFR amplification was identified in 4 (29%) of 14 cases of primary penile SCC and lymph node metastases [12]. In fact, a small study of 30 patients has suggested that EGFR expression has been found to be strongly related to increased risk of recurrence and poorer prognosis in patients with penile SCC [13]. Given that EGFR overexpression is common and associated with poor prognosis, it is a therapeutic target in systemic penile cancer treatment.

Therapy targeted towards EGFR includes monoclonal antibodies cetuximab and panitumumab. For patients with tumors overexpressing EGFR, there are previous reports showing promising clinical benefit with EGFR inhibitor treatment [7]. In a retrospective study of 17 patients treated with cetuximab either alone or with cisplatin, there were 4 (23.5%) partial responses. Another study with 28 patients receiving anti-EGFR drugs such as cetuximab, panitumumab, and nimotuzumab had 50% of patients showing a response to treatment. 15 of 28 patients were receiving anti-EGFR therapy as second-line therapy. Overall, 50% of the patients showed response to treatment with median PFS of 3 months [7].

In addition to anti-EGFR therapy, immune checkpoint inhibition with PD-1 and PD-ligand 1 (PD-L1) is an important therapeutic target for penile cancer among other malignancies. Udager et al. found that 23 (62.2%) of 37 primary penile SCC tumors were positive for PD-L1 expression. Furthermore, they found significant association between PD-L1 expression and regional lymph node metastasis as well as decreased cancer-specific survival [9]. Another study by Cocks et al. found 21 (40%) of 53 penile SCC tumors expressing PD-L1 [14]. These small studies suggest rational for checkpoint inhibitors as therapeutic targets. Pembrolizumab and nivolumab are two monoclonal antibodies against PD-1 that are FDA-approved in other cancers, such as melanoma. Atezolizumab and durvalumab are two drugs of monoclonal antibodies against PD-L1 that are FDA-approved for urothelial and non-small-cell lung cancer. Avelumab, a PD-L1 inhibitor, is approved for metastatic Merkel cell carcinoma. There are ongoing clinical trials studying the use of pembrolizumab and nivolumab in penile SCC.

In this case series, we describe two patients who developed metastatic penile cancer, which progressed after surgery and chemotherapeutic approach. However, while there were no clinical trials available for their treatment at the time of progression, they were treated with EGFR inhibitors and immune checkpoint inhibitors with compassionate use that prolonged their survival. The first patient had an additional 12 months of survival after starting anti-EGFR therapy upon failing primary chemotherapy. This was significant given that tumor progression beyond primary chemotherapy has been noted to have median overall survival (OS) of less than six months. It is imperative to include patients with rare tumors such as penile cancer in the multiple ongoing basket trials that are running in most institutions with the hope to offer novel therapies to this patient population.
